# Enhanced Humoral Immune Response After COVID-19 Vaccination in Elderly Kidney Transplant Recipients on Everolimus Versus Mycophenolate Mofetil–containing Immunosuppressive Regimens

**DOI:** 10.1097/TP.0000000000004177

**Published:** 2022-05-11

**Authors:** Silke E. de Boer, Stefan P. Berger, Coretta C. van Leer–Buter, Bart-Jan Kroesen, Debbie van Baarle, Jan-Stephan F. Sanders

**Affiliations:** 1 Department of Internal Medicine, Division of Nephrology, University Medical Center Groningen and University of Groningen, Groningen, The Netherlands.; 2 Department of Medical Microbiology (Clinical Virology), University Medical Centre Groningen and University of Groningen, Groningen, The Netherlands.; 3 Department of Laboratory Medicine, University Medical Centre Groningen and University of Groningen, Groningen, The Netherlands.; 4 Department of Medical Microbiology and Infection Prevention, University Medical Center Groningen and University of Groningen, Groningen, The Netherlands.

## Abstract

**Background.:**

Elderly kidney transplant recipients (KTRs) represent almost one third of the total kidney transplant population. These patients have a very high coronavirus disease 2019 (COVID-19)–related mortality, whereas their response to COVID-19 vaccination is impaired. Finding ways to improve the COVID-19 vaccination response in this vulnerable population is of uttermost importance.

**Methods.:**

In the OPTIMIZE trial, we randomly assign elderly KTRs to an immunosuppressive regimen with standard-exposure calcineurin inhibitor (CNI), mycophenolate mofetil, and prednisolone or an adapted regimen with low dose CNI, everolimus, and prednisolone. In this substudy, we measured the humoral response after 2 (N = 32) and 3 (N = 22) COVID-19 mRNA vaccinations and the cellular response (N = 15) after 2 vaccinations.

**Results.:**

. The seroconversion rates of elderly KTRs on a standard immunosuppressive regimen were only 13% and 38% after 2 and 3 vaccinations, respectively, whereas the response rates of KTRs on the everolimus regimen were significantly higher at 56% (*P* = 0.009) and 100% (*P* = 0.006). Levels of severe acute respiratory syndrome coronaVirus 2 IgG antibodies were significantly higher at both time points in the everolimus group (*P* = 0.004 and *P* < 0.001). There were no differences in cellular response after vaccination.

**Conclusions.:**

An immunosuppressive regimen without mycophenolate mofetil, a lower CNI dose, and usage of everolimus is associated with a higher humoral response rate after COVID-19 vaccination in elderly KTRs after transplantation. This encouraging finding should be investigated in larger cohorts, including transplant recipients of all ages.

## INTRODUCTION

Kidney transplantation in elderly recipients is rapidly increasing to almost 30%.^1,2^ The coronavirus disease 2019 (COVID-19)–associated mortality risk in this population is very high: approximately 23% for kidney transplant recipients (KTRs) of 65 y and increasing with 3.7% for every 10 y of age.^3^

Vaccination is a very effective way to prevent a severe course of COVID-19 infection in the general population.^4,5^ Unfortunately, several studies have shown that the seroconversion rates after 2 COVID-19 mRNA vaccinations are relatively low in KTRs, with percentages varying between 4% and 57%.^6,7^ Next to the use of mycophenolate mofetil (MMF) as a major factor associated with poor seroresponse, the response rates after COVID-19 mRNA vaccination also decrease with increasing age.^7,8^ Hence, finding ways to improve the COVID-19 vaccination response for this vulnerable population of elderly KTRs is of utmost importance.

In the current study, we, therefore, addressed the hypothesis that elderly KTRs on an immunosuppressive treatment without MMF but including the mammalian target of rapamycin (mTOR) inhibitor everolimus (EVR) have a better seroresponse after COVID- 19 mRNA vaccination than KTRs on immunosuppressive treatment with MMF.

## MATERIALS AND METHODS

### Patient Population

We measured the humoral immune response after 2 COVID-19 vaccinations in 32 elderly KTRs included in the ongoing OPTIMIZE trial (ClinicalTrials.gov Identifier: NCT03797196) at the University Medical Center Groningen. We also measured the cellular response in 15 of these KTRs. Furthermore, we measured the humoral response after the third COVID-19 vaccination in 22 of these 32 KTRs. The OPTIMIZE trial and this substudy have been approved by the Medical Research Ethical Committee of the University Medical Center Groningen (2018.698) and are in line with the ethical principles laid down in the Declaration of Helsinki, Brazil, October 2013. The design of this study has been described in detail before.^9^

In short, the OPTIMIZE trial is an open-label, randomized, multicenter, clinical trial that includes the elderly (≥65 y), de novo KTRs. KTRs are randomized to an immunosuppressive regimen with standard-exposure calcineurin inhibitor (CNI), MMF, and prednisolone (the MMF group) or a regimen with low dose CNI, EVR, and prednisolone (the EVR group). For the MMF group, initial tacrolimus target trough levels are 8 to 12 ng/mL, tapered to 6 to 10 from 3 mo onward, and 5 to 8 ng/mL from 6 mo after transplantation. MMF is given in a dose of 500 mg bid throughout the trial. For the EVR group, the initial tacrolimus target trough level is 5 to 7, tapered to 2 to 4 ng/mL from 3 mo onwards, and 1.5 to 4 ng/mL from 6 mo after transplantation. EVR target trough level is 3 to 6 ng/mL throughout the trial. All KTRs receive induction therapy with basiliximab. Alternative induction therapy with T-cell–depleting agents is not permitted.

For the current study, we enrolled only KTRs included in the OPTIMIZE trial at the University Medical Center Groningen (N = 76). All patients received COVID-19 vaccination as part of routine patient care within the Dutch national COVID-19 vaccination program.

**FIGURE 1.**
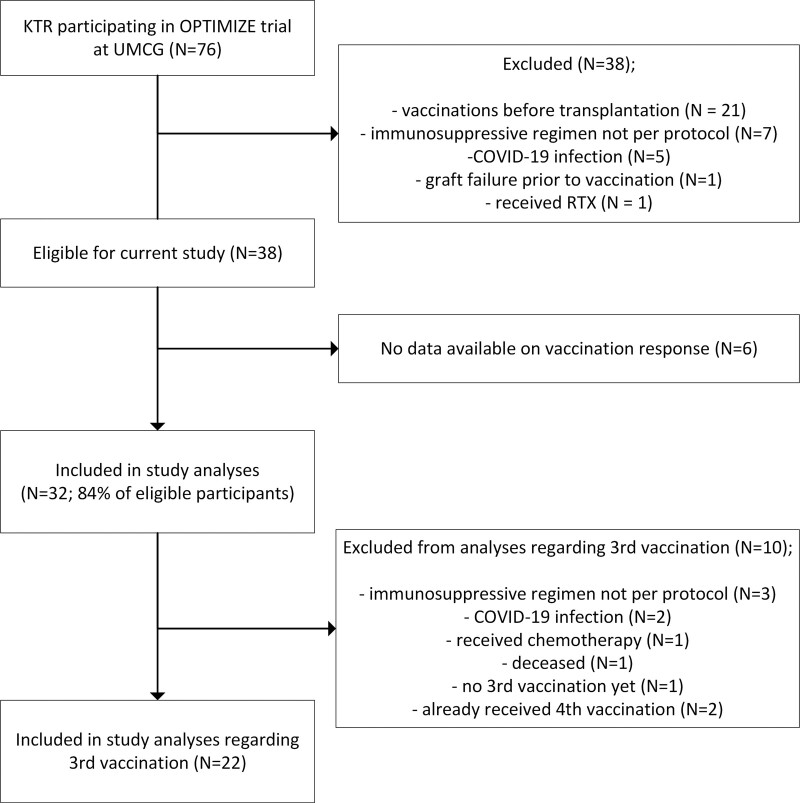
Patient flow diagram. COVID-19, coronavirus disease 2019; KTR, kidney transplant recipient; RTX, rituximab; UMCG, University Medical Center Groningen.

We enrolled 32 OPTIMIZE participants who had not been previously tested positive for severe acute respiratory syndrome coronaVirus 2 (SARS-CoV-2) by polymerase chain reaction, who underwent vaccination against COVID-19, and who were treated with the immunosuppressive regimen per protocol. A total number of 38 participants were eligible for the current study, but no data on vaccination response were available for 6 participants. See Figure 1 for the patient flow diagram. For 22 KTRs, we also measured the humoral response after the third COVID-19 vaccination. For the remaining 10, analysis was not possible or meaningful (Figure 1). For 18 KTRs, blood samples for isolation of peripheral blood mononuclear cells (PBMCs) were taken and used for analysis of the cellular response after 2 vaccinations. Three samples could not be used for quality reasons, so a total of 15 samples were analyzed.

### SARS-CoV-2 Anti–Spike Receptor Binding Domain IgG Antibody

The SARS-CoV-2 IgG II Quant ELISA method (Architect, Abbott, IL, USA), a chemiluminescent microparticle immunoassay, was used for the quantitative determination of IgG antibodies against the spike receptor-binding domain of SARS-CoV-2.^10^ Testing of serum and plasma samples was done according to the manufacturer’s instructions. Results are expressed in arbitrary units (AU)/mL, with 50 AU/mL as a positive cutoff and a maximal threshold of quantification of 40 000 AU/mL. As primary outcome measure, KTRs were either classified as responder (IgG antibody concentration ≥50 AU/mL) or nonresponder (IgG antibody concentration <50 AU/mL).

### T-lymphocyte Reactivity Against Spike Protein

When PBMCs were available, the SARS-CoV-2–specific T-lymphocyte response was measured after stimulation of PBMCs isolated from heparinized venous blood obtained after the second vaccination. The number of interferon-gamma (IFN-ɣ)–producing T lymphocytes after stimulation with SARS-CoV-2 spike overlapping peptide pools was assessed using an IFN-ɣ enzyme-linked immune adsorbent spot (ELISpot) assay. SARS-CoV-2 S1 and S2 peptide pools (JPT Peptide Technologies), consisting of 15-mer peptides overlapping 11 amino acids that cover the entire sequence of the viral proteins, were used for stimulation of the PBMCs in a concentration of 0.5 µg/mL. Dimethyl sulfoxide (0.4%; Sigma) was used as a negative control and phytohemagglutinin (Remel Europe Ltd; 4 µg/mL) as a positive control. Spot forming cells (SFC) were quantified with the AID ELISpot/Fluorospot reader and calculated to SFCs/10^6^ PBMCs. The average of the dimethyl sulfoxide negative control was subtracted per stimulation. To define the total spike-specific SFC, the SFCs of the separate S1 and S2 peptide pools were summed.

Results are expressed in number of IFN-ɣ spots per 10^6^ PBMCs. More than 50 IFN-ɣ spots per 10^6^ PBMCs was considered a positive response.^11^

### Statistical Analysis

Normally distributed data are presented as mean ± SD. Nonnormally distributed data are presented as median (interquartile range).

Categorical data are presented in numbers (percentages). Differences between the MMF and EVR group for normally and nonnormally distributed data were assessed by unpaired *t* test and Mann-Whitney test, respectively. Differences for binary variables were assessed by χ^2^ test or Fisher exact test, if applicable.

Data were analyzed using SPSS software, version 23.0, and GraphPad Prism 8.4.2. In all analyses, *P* < 0.05 was considered to indicate a statistically significant difference.

## RESULTS

### Baseline Characteristics

Baseline characteristics of the study population (at the time of the first vaccination) are shown in Table 1. The mean age of the participants was 72 ± 4 y, and 12 (38%) were female. Median estimated glomerular filtration rate was 42 (32–56) mL/min/1.73 m^2^. All but 1 participant were not transplanted before the current transplantation. Median time between transplantation and first vaccination was 32 (18–43) wk. None of the KTRs was treated for rejection. Most of the clinical characteristics did not differ significantly between both treatment groups, but there were more KTRs with a living donor in the EVR group (*P* = 0.043) and there were more preemptive procedures in the EVR group (*P* = 0.037). Most participants (21) received 2 mRNA-1273 vaccinations (Moderna Biotech Spain, SL); the other 11 participants received 2 mRNA-BNT162b2 vaccinations (COMIRNATY, Pfizer-BioNTech). Median interval between first and second vaccination was 28 d (28–35). Median time between the second vaccination and the response measurement was 40 d (28–64). Type of vaccination and time frames did not differ between groups (Table 2).

**TABLE 1.** Baseline characteristics

Variable	All (N = 32)	MMF group (N = 16)	EVR group (N = 16)	*P*
Female, n (%)	12 (38)	7 (44)	5 (31)	0.465
Caucasian, n (%)	29 (91)	16 (100)	13 (81)	0.226
Age, y, mean (SD)	72 ± 4	72 ± 4	71 ± 5	0.283
BMI, kg/m^2^, mean (SD)	27.4 ± 3.5	27.1 ± 3.7	27.7 ± 3.3	0.617
Number of comorbidities, median (IQR)	2 (1–3)	2 (1–3)	2 (1–3)	0.800
Comorbidities				
Hypertension, n (%)	31 (97)	15 (94)	16 (100)	1.000
Diabetes mellitus, n (%)	15 (47)	7 (44)	8 (50)	1.000
History of coronary artery disease, n (%)	11 (34)	4 (25)	7 (44)	0.264
Heart failure, n (%)	4 (13)	2 (13)	2 (13)	1.000
Chronic lung disease, n (%)	5 (16)	2 (13)	3 (19)	1.000
History of malignancy,^a^ n (%)	4 (13)	3 (19)	1 (6)	0.600
Autoimmune disease, n (%)	5 (16)	4 (25)	1 (6)	0.333
Primary diagnosis				0.702
Unknown, n (%)	2 (6)	0	2 (13)	–
Glomerulonephritis, n (%)	4 (13)	3 (19)	1 (6)	–
Interstitial nephritis, n (%)	4 (13)	2 (13)	1 (6)	–
Cystic kidney disease, n (%)	4 (13)	3 (19)	1 (6)	–
Other congenital and hereditary kidney disease, n (%)	0	0	0	–
Renal vascular disease. excluding vasculitis, n (%)	12 (38)	5 (31)	7 (44)	–
Diabetes mellitus, n (%)	3 (9)	1 (6)	3 (13)	–
Other multisystem diseases, n (%)	2 (6)	1 (6)	1 (6)	–
Other	1 (3)	1 (6)	0	–
Transplant characteristics				
First kidney transplant, n (%)	31 (97)	15 (94)	16 (100)	1.000
Donor type				
Living, n (%)	5 (16)	0	5 (31)	0.043^b^
DBD donor, n (%)	9 (28)	6 (38)	3 (17)	0.692
DCD donor, n (%)	18 (56)	10 (63)	8 (50)	
Preemptive, n (%)	8 (25)	1 (6)	7 (44)	0.037^b^
Cyclosporine (instead of tacrolimus), n (%)	4 (13)	3 (19)	1 (6)	0.600
eGFR, mL/min/1.73m^2^, median (IQR)^c^	42 (33–53)	39 (33–52)	47 (33–58)	0.381
Rejection treatment, n	0	0	0	–

a Including melanomas, excluding all other skin malignancies.

b *P* < 0.05 for significance.

c eGFR at time of first vaccination.

BMI, body mass index; DBD, donation after brain death; DCD, donation after circulatory death; eGFR, estimated glomerular filtration rate; EVR, everolimus; IQR, interquartile range; MMF, mycophenolate mofetil.

**TABLE 2.** Vaccination-related characteristics

Variable	All (N = 32)	MMF group (N = 16)	EVR group (N = 16)	*P*
First 2 vaccinations with mRNA-1273 vaccine, n (%)	21 (66)	11 (69)	10 (63)	0.602
Time between KTX and first vaccination, wk, median (IQR)	32 (18–43)	28 (20–45)	34 (17–42)	0.850
Time between first and second vaccination, d, median (IQR)	28 (28–35)	28 (28–35)	28 (28–35)	0.395
Time between second vaccination and humoral response measurements, d, median (IQR)	40 (28–64)	42 (30–62)	36 (24–77)	0.806
	(range 12–180)	(range 12–117)	(range 22–180)	
Responder (humoral) after 2 vaccinations, n (%)	11 (34)	2 (13)	9 (56)	0.009^a^
KTRs with measurement after third vaccination, n (%)	22 (69)	13 (81)	9 (56)	0.127
Third vaccination with mRNA-1273 vaccin, n (%)	4 (18)	3 (23)	1 (11)	0.616
Time between second and third vaccination, d, median (IQR)	192 (166–207)	180 (159–202)	202 (184–215)	0.082
Time between third vaccination and humoral response measurements, d, median (IQR)	61 (44–83)	62 (27–77)	54 (49–86)	0.841
Responder (humoral) after 3 vaccinations, n (%)	14 (64)	5 (38)	9 (100)	0.006^a^

a *P* < 0.05 for significance.

EVR, everolimus; IQR, interquartile range; KTR, kidney transplant recipient; KTX, kidney transplantation; MMF, mycophenolate mofetil.

### Humoral Response After 2 Vaccinations

**FIGURE 2.**
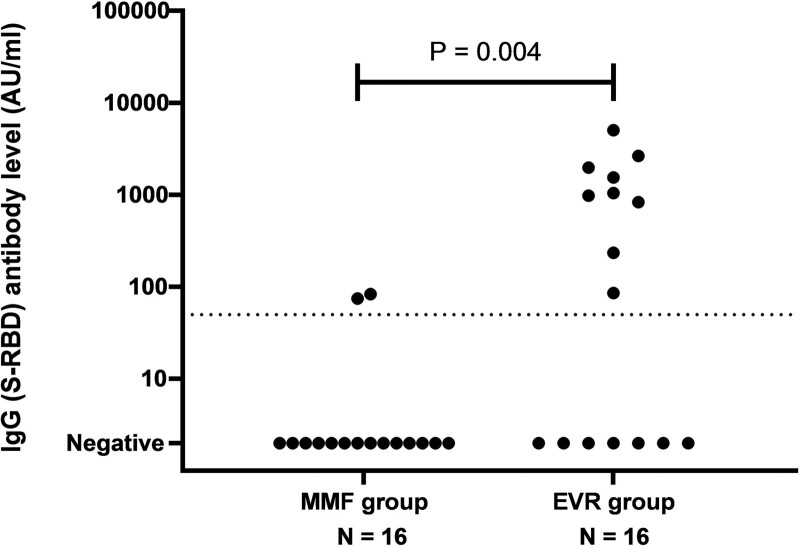
IgG (spike receptor-binding domain [S-RBD]) antibody level after 2 vaccinations. The dotted line indicates threshold for seroresponse. Maximal threshold of quantification is 40 000 AU/mL. AU, arbitrary units; EVR, everolimus; MMF, mycophenolate mofetil.

In 32 participants in total, IgG antibody levels were measured: 16 in both the MMF and EVR group. Two participants in the MMF group were classified as responders after 2 vaccinations (13%) versus 9 participants in the EVR group (56%) (*P* = 0.009). Also, the IgG antibody levels were significantly higher in the EVR group (160 AU/mL [0–142]) than in the MMF group (0 AU/mL [0–0]) (*P* = 0.004) (Figure 2).

### Humoral Response After 3 Vaccinations

In 22 participants, 13 in the MMF group and 9 in the EVR group, the humoral response after a third COVID-19 vaccination was measured. Most KTRs (82%) received RNA-BNT162b2 as a third COVID-19 vaccine. Median time between the second and third vaccination was 192 d (166–207), and median time between the third vaccination and response measurement was 61 d (44–83). Type of vaccine and time frames did not differ between groups (Table 2).

In the MMF group, the third vaccination led to an increase in IgG antibody levels in 5 participants (38%), and of the 12 seronegative patients, 4 became seropositive. In the EVR group, all patients showed an increase in IgG antibody levels, and the 3 seronegative patients all became seropositive.

**FIGURE 3.**
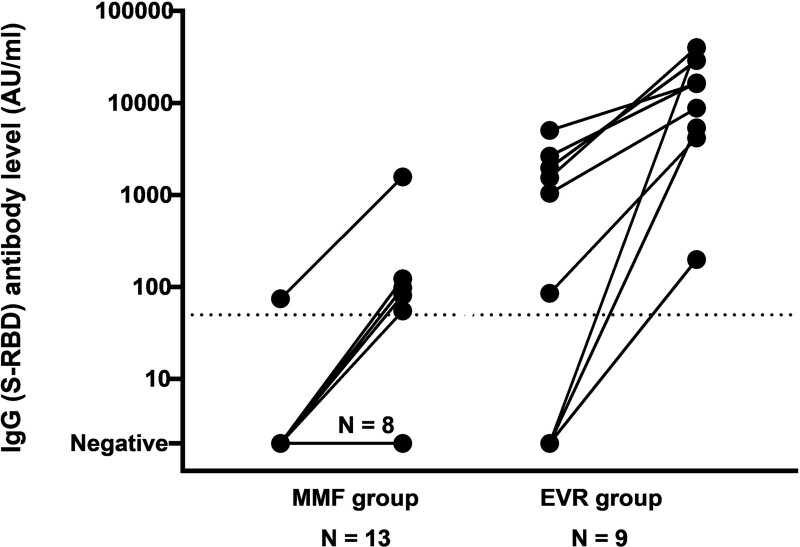
Changes in IgG (spike receptor-binding domain [S-RBD]) antibody level between second and third vaccination. The dotted line indicates threshold for seroresponse. Maximal threshold of quantification is 40 000 AU/mL. AU, arbitrary units; EVR, everolimus; MMF, mycophenolate mofetil.

After the third vaccination, a total of 5 out of 13 participants in the MMF could be classified as responders (38%), whereas all (9/9) participants in the EVR group could be classified as responders (100%) (*P* = 0.006). The IgG antibody levels were also significantly higher in the EVR group than in the MMF group (16 288 (4782–34 472) vs 0 (0–90)) (*P* = 0.006) (Figure 3).

### Cellular Response After 2 Vaccinations

For 6 KTRs in the MMF group and 9 KTRs in the EVR group, the T-cell response against SARS-CoV-2 was measured at a median duration of 104 (54–146) d after the second vaccination. In the MMF group, the number of KTRs with a positive T-cell response was 3 (50%) versus 4 (44%) in the EVR group (*P* = 1). One (17%) of the 6 KTRs in the MMF group had both a humoral and a cellular response versus 3 out of 9 in the EVR group (*P* = 0.604). The number of IFN-ɣ spots per 10^6^ PBMCs did not differ between groups (49 (33–212) in the MMF group vs 47 (14–98) in the EVR group) (*P* = 0.814) (Figure S1, SDC, http://links.lww.com/TP/C431). There was no association between the presence of a positive T-cell response and the IgG antibody level (*P* = 0.807).

## DISCUSSION

In this analysis within an ongoing randomized, controlled study, we found that the seroresponse rates after 2 and 3 COVID-19 vaccinations were higher in elderly KTRs on an immunosuppressive regimen consisting of reduced-dose CNI with EVR and prednisolone than on a standard CNI-based immunosuppressive regimen with MMF and prednisolone. Next to the increased seroresponse rate, the EVR group also showed markedly higher IgG antibody levels.

The response rate of 56% in the EVR group after 2 vaccinations should be considered as a relevant and important finding, especially when viewed in the context of the following perspective: Overall, humoral response rates in different studies after 2 COVID-19 mRNA vaccinations for KTRs vary between 4% and 57%.^6,7^ The highest percentage of 57% was found in one of the largest studies thus far, the Dutch REnal patients COVID-19 VACcination immune response study^7^; however, the participants in the current study were elderly, were on a triple immunosuppressive regimen, and had a relatively short time after transplantation. All these factors have previously been associated with lower humoral response rates after COVID-19 vaccination.^7^ Ergo, the seroresponse rate in the EVR group equals or exceeds the seroresponse rates so far reported. This seroresponse rate in the EVR group is in stark contrast with the low seroresponse rate of only 13% in the MMF group, which reflects the accumulation of risk factors in this high-risk population.

Although no such direct comparison in a randomized study has been made before, several studies have shown that KTRs on MMF or mycophenolic acid are less likely to have an effective humoral immune response after 2 COVID-19 vaccinations.^7,12,13^ Likewise, seroresponse to influenza vaccination in KTRs is weaker for KTRs on MMF than for those who are not.^14,15^ Previously, a study compared humoral immune responses after 3 different non–COVID-19 vaccinations (immunocyanin, pneumococcal polysaccharide, and tetanus toxoid) for KTRs on different immunosuppressive regimens. In KTRs on prednisolone and EVR, primary and secondary humoral immune responses were preserved, whereas these responses were absent in KTRs on prednisolone and mycophenolic acid.^16^ Also, better humoral response rates after 2 COVID-19 vaccinations for KTRs on mTOR inhibitors have been described before.^7,12,17,18^

Recently, a third COVID-19 mRNA vaccination has been shown to be effective and safe in several studies in KTRs,^17,19,20^ including a randomized, clinical trial, increasing seroresponse by approximately 43% to 55%^20^; however, also in these studies, a significant percentage of KTRs remained seronegative, and they are in striking contrast with the promising results of our randomized study, showing that all EVR-treated patients were seropositive after 3 COVID-19 vaccinations.

These data warrant further research into the effects of an EVR-containing immunosuppressive regimen on the (COVID-19) vaccination response and clinical outcomes of COVID infection in vaccinated organ transplant recipients on EVR.

When considering the above, it might be hypothesized that the difference in vaccination response between the EVR group and the MMR group is not solely explained by the absence of MMF in the EVR group. We hypothesize that, although the absence of MMF likely plays an important role, there are 2 more reasons that the EVR group shows a better response. The first one is the difference in CNI target trough levels between both groups, as high CNI levels are associated with lower humoral immune response rates.^8^ The second possible reason is the presence of an mTOR inhibitor. In a randomized, observer-blind, placebo-controlled trial study in elderly (≥65 y) volunteers without unstable medical conditions treated with EVR, an enhanced response to influenza vaccination by about 20% was shown.^21^ The proposed mechanism was the decrease in programmed death-1 (PD-1)–positive CD4^+^ and CD8^+^ T cells found in EVR-treated patients, compared with placebo-treated patients. PD-1–positive CD4^+^ and CD8^+^ T cells accumulate with age and have diminished responses to antigen stimulation.^21^ Hence, lower percentages of PD-1–positive CD4 and CD8 T cells may contribute to enhanced immune function and improve the quality of T-cell responses to antigenic stimulation in the elderly and thereby also promote better humoral immunity.

Additionally, the recently published TRANSplant eFficacy and safety Outcomes with an eveRolimus-based regiMen (TRANSFORM) trial, which included 2037 de novo KTRs, showed that treatment with EVR and reduced-exposure CNI led to a reduced risk of infectious complications, whereas EVR and reduced CNI were noninferior to treatment with mycophenolate with standard-exposure CNI regarding rejection risk.^22^

Despite the proposed mechanism of decreased PD-1+ CD4 and CD8 T cells in an immunosuppressive regimen with EVR, we did not detect a difference between both treatment groups regarding the cellular response as measured by ELISpot. A recent observational study showed that KTRs on mTOR inhibitors were more likely to have a T-cell response than KTRs not on mTOR inhibitors.^18^ This difference with our results is possibly explained by the smaller numbers in our study. Also, methods differed between the studies. Whereas we used a sensitive ELISpot assay, which is not affected by in vitro activity of immunosuppressive drugs, an IFN-ɣ release assay was used in the study described by Netti et al.^18^

Finally, more in-depth studies into additional functional and phenotypic characteristics of the T cells may reveal differences between regimes we now could not observe.

The main strength of this study is that we were able to measure the vaccination seroresponse after 2 and 3 vaccinations in a vulnerable, randomized population that did not significantly differ, apart from their immunosuppressive regimen. The study also has a few limitations that need to be mentioned. The study size was limited. Nevertheless, we found highly significant differences. Second, we only measured the cellular response in a part of the participants. Third, in this study, we did not measure virus-neutralizing antibodies; however, we previously found a significant correlation between the S1-specific IgG antibody level and the titer of virus-neutralizing antibodies.^7^ Higher levels of not only virus-neutralizing antibodies but also IgG antibody levels have been reported to be associated with a lower risk of COVID-19.^23-25^ This implies that KTRs in the EVR group might be at lower risk for symptomatic COVID-19 than elderly patients in the MMF group.

To conclude, we describe seroresponse rates after 2 and 3 COVID-19 vaccinations in a randomized population of elderly KTRs of, respectively, 56% and 100% for KTRs on an EVR-containing immunosuppressive regimen and only 13% and 38% for KTRs on an MMF-containing immunosuppressive regimen. Our results are encouraging because elderly KTRs are at high risk for both COVID-19–related mortality and a poor response to COVID-19 vaccination.

## ACKNOWLEDGMENTS

The OPTIMIZE study group consist of Sanders, Jan-Stephan F., MD, PhD, Department of Internal Medicine, Division of Nephrology, University Medical Center Groningen, University of Groningen, Groningen, The Netherlands; Berger, Stefan P., MD, PhD, Department of Internal Medicine, Division of Nephrology, University Medical Center Groningen, University of Groningen, Groningen, The Netherlands; Bemelman, Frederike J., MD, PhD, Department of Internal Medicine, Division of Nephrology, Amsterdam Universal Medical Center, Amsterdam, The Netherlands; Betjes, Michiel G.H., MD, PhD, Department of Internal Medicine, Division of Nephrology & Transplantation, Erasmus MC, Erasmus University Medical Center, Rotterdam, The Netherlands; Hesselink, Dennis A., MD, PhD, Department of Internal Medicine, Division of Nephrology & Transplantation, Erasmus MC, Erasmus University Medical Center, Rotterdam, The Netherlands; Hilbrands, Luuk B., MD, PhD, Department of Internal Medicine, Division of Nephrology, Radboud University Medical Center, Nijmegen, The Netherlands; Kuypers, Dirk R.J., MD, PhD, Department of Nephrology and Renal Transplantation, University Hospitals Leuven, Leuven, Belgium; Nurmohamed, S. Azam., MD, PhD, Department of Internal Medicine, Division of Nephrology, Amsterdam Universal Medical Center, Amsterdam, The Netherlands; de Vries, Aiko P.J., MD, PhD, Department of Internal Medicine, Division of Nephrology; and Leiden Transplant Center, Leiden University Medical Center, Leiden University, Leiden, The Netherlands; and van Zuilen, Arjan D., MD, PhD; Department of Internal Medicine, Division of Nephrology, University Medical Center Utrecht, Utrecht, The Netherlands.

## Supplementary Material


