# Supplementary material for: Enhanced Humoral Immune Response After COVID-19 Vaccination in Elderly Kidney Transplant Recipients on Everolimus Versus Mycophenolate Mofetil–containing Immunosuppressive Regimens
Source: Transplantation. 2022 May 11;106(8):1615–21. doi: 10.1097/TP.0000000000004177 (PMC9311282; doi:10.1097/TP.0000000000004177)
Supplement: Supplementary file 1 [file tp-106-1615-s001.pdf]

Figure S1: IFN-ɣ spots per 10^6 PBMCs after 2 vaccinations

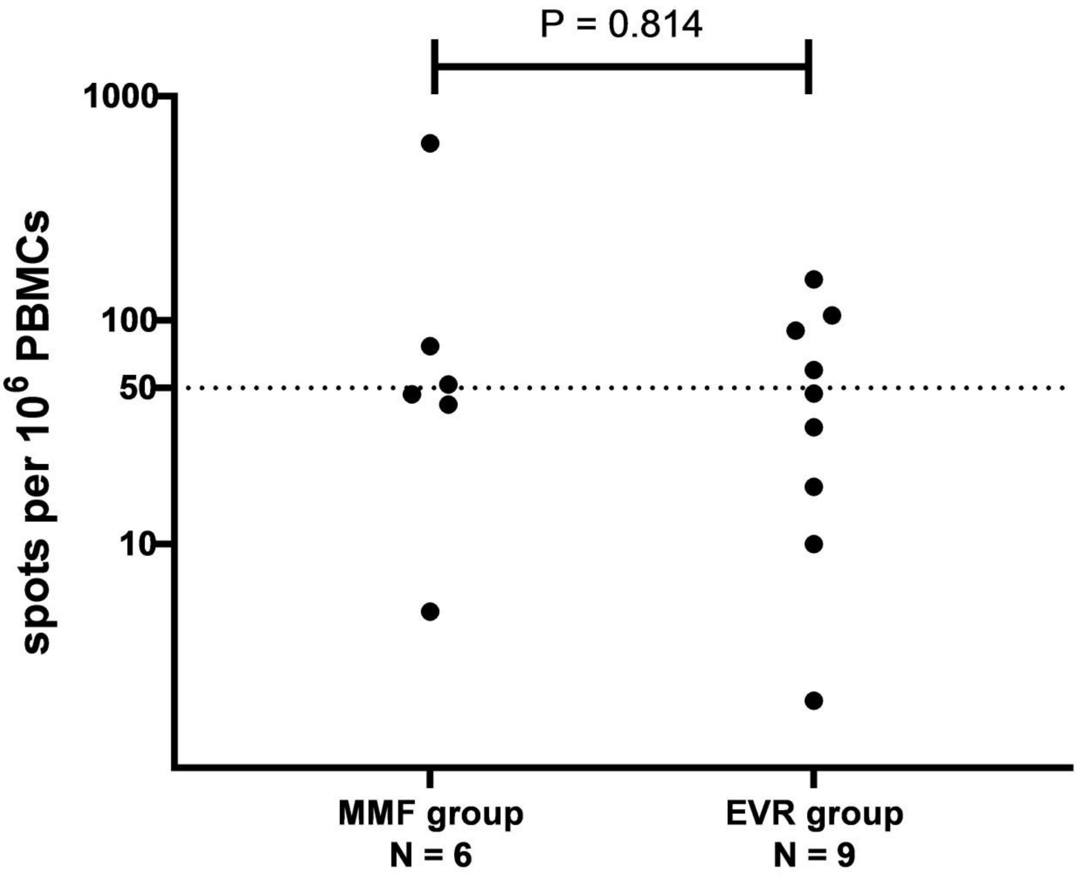

Dotted line indicates threshold for positive T-cell response.
